# A Closer Look at GE Corn Findings

**DOI:** 10.1289/ehp.120-a421

**Published:** 2012-11-01

**Authors:** Wendee Nicole

**Affiliations:** Wendee Nicole, based in Houston, TX, has written for *Nature*, *Scientific American*, *National Wildlife*, and other magazines.

A long-term animal toxicity study claiming adverse effects of genetically engineered (GE) corn^1^ has caused an international maelstrom. The study examined how exposure to the herbicide Roundup® and NK603 Roundup Ready® maize (GE corn engineered to withstand glyphosate, the active ingredient in Roundup) affected Sprague-Dawley rats over a 2-year period, roughly equivalent to 65 years in humans.^2^ Images from the paper of enormous tumors in the rats have riveted the public, yet researchers and industry have raised numerous concerns about the design of the study, which was led by Gilles-Eric Séralini, a molecular biology professor at the University of Caen in France. The authors also came under fire for insisting journalists sign an agreement preventing contact with third parties prior to publication, an unheard-of embargo term,^3^ which they claimed would prevent leaks of the sensitive paper.^4^

Upon publication of the article, the European Commission asked the European Food and Safety Agency (EFSA) to assess whether the findings warranted revisiting earlier safety assessments of NK603. In its assessment EFSA determined the study design and statistical analysis to be of insufficient quality for use in food safety risk assessment.^5^ The agency concluded by inviting the authors to provide additional documentation so the study could be assessed more thoroughly, an invitation that had not been accepted when this article went to press. In a separate review, the German Federal Institute for Risk Assessment assessed the glyphosate findings specifically and concluded that the main findings were not supported by the data.^6^

The investigators studied a total of 100 male and 100 female rats, with each sex divided into 10 treatment groups of 10 animals each. Three groups of each sex were given feed supplemented with different doses of NK603 that had been grown with Roundup, three were given feed containing untreated NK603, and three were given a standard diet but water containing Roundup. One control group for each sex was fed a standard diet and plain water. The team examined the rats twice weekly, sampled blood and urine throughout the study, and conducted a histologic examination of nine different organs.

According to the authors, females in all treatment groups suffered higher mortality rates than controls as a result of tumors forming sooner and more often, with Roundup-only animals developing the most tumors. By the final month, they wrote, 50–80% of treated females had visible tumors, compared with only 30% of controls; 93% of these tumors were in the mammary gland.

Treated males reportedly developed severe liver and kidney damage more often than controls. Additionally, the investigators claimed to find an association between Roundup exposure and increased cytochrome activity, between NK603 and reduced transcription rates, and between NK603 combined with Roundup and increased smooth endoplasmic reticulum. They wrote that mortality and tumorigenesis did not show a linear dose–response relationship with most treatments, but peaked at lower doses, suggesting the non-monotonic dose–response curve found with endocrine disruptors.^7^

Numerous independent researchers have criticized several aspects of the study. Important details were not shown: food and water intake, dietary composition, an explanation of why controls died, and images of control tumors. Perhaps most damaging is the lack of statistical analysis of the mortality data and corrections for multiple comparisons. “What this study does *not* show is that exposing these rats to [GE] corn and/or Roundup makes any difference to the frequency of cancers or other diseases. It cannot because no statistical tests have been applied,” says Agnès Ricroch, a lecturer in evolutionary genetics and plant breeding at AgroParisTech and a researcher at the University of Orsay. “Omission of statistical analysis is inexcusable.”

Others criticized the small size of the groups, as well as the comparison of nine treatment groups to the same control animals. “They looked at two hundred animals with only twenty controls,” says Michael Eisen, an investigator at Howard Hughes Medical Institute. “Because of all the conditions . . . you don’t undercut the control. The experiment was designed to get a significant result against [NK603].”

But the authors say their study was intended to extend the 90-day trials conducted by Roundup and NK603 manufacturer Monsanto^8^—which formed the basis of past safety assessments of the corn—over a longer period to better assess long-term exposure. Although many criticized the use of tumor-prone Sprague-Dawley rats, these animals “represent a human equivalent model regarding the most frequent tumors,” says Fiorella Belpoggi, director of the Cesare Maltoni Cancer Research Centre of the Ramazzini Institute.^9^

Theo Colborn, president of The Endocrine Disruption Exchange, says of the study, “By taking compliance-level protocols . . . and embellishing them, this study moved to a new level of research.” And in an essay published online, Corinne Lepage, founder of the Committee for Research and Independent Information on Genetic Engineering—the nongovernmental organization that partially funded the study—wrote that Seralini’s 2-year study “proves that 90-day studies cannot show anything as the first tumours did not start to show until the fourth or fifth month.”^3^

Indeed, the observation that tumors first developed at 4 months, with most appearing after 18 months, does allude to ongoing concerns about the lack of mandatory premarket long-term testing of GE foods. “It certainly suggests we ought to do longer-term feeding studies,” says Ted Schettler, science director of the Science and Environmental Health Network. “A ninety-day feeding study is just not adequate.” The problem, investigators largely agree, is that the flaws in the study’s design and reporting render the data impossible to interpret.

Time will tell whether the study’s findings can be replicated. Meanwhile, perhaps a lesson can be learned from this experience. “If small studies on transgenic crops imply risk or harm, an additional level of rigor must be employed to ensure reproducibility and hard significance of the primary findings,” says Kevin M. Folta, an associate professor in the University of Florida Horticultural Science Department. “[I]f a report has a potential to impact seventy percent of food products in the USA, it needs to be sterling in methods and interpretations.”
